# Exposing the primary failure of gender-affirming care: Response to Erasmus (2024)

**DOI:** 10.1177/10398562241291952

**Published:** 2024-10-13

**Authors:** Andrew J Amos

Dear Editor,

I agree with Erasmus^1^ there is an apparent contradiction between the position that the gender-affirming model of care (GAMOC) ‘assumes there are no pathological aetiologies of gender diversity and protects this assumption by forbidding the assessment of pathology in individual patients’ and the recommendation that gender clinicians ‘exclude other possible causes of apparent gender incongruence prior to the initiation of gender-affirming treatment’. However, this doesn’t reflect an error in my analysis^2^ but identifies the fundamental failure in the World Professional Association for Transgender Health (WPATH) endorsed Standards of Care (SoC) which makes them incompatible with ethical and competent medical care.^2^

As my article states, no evidence or theory has ever been put forward to support the SoC assertion that ‘[g]ender diversity is a natural variation in people and is not inherently pathological’ (pS34).^2^ Presumably Erasmus is aware of this as he does not point to any such evidence or theory. In addition, the SoC do not recommend the GAMOC as a treatment for a medical diagnosis such as gender dysphoria or gender incongruence. They recommend that it be offered as a human right to people who report gender diverse identities. Unlike medical diagnoses, gender diversity is a loose category determined by the subjective report of patient experiences with no fixed characteristics, which vary in undefined ways, and includes such self-evidently pathological states as the desire for castration.^2^

As a result, in the model described by the SoC there is no inherent conflict between the diagnosis of a psychotic illness, or indeed any severe psychiatric illness, and the GAMOC, because the latter is contingent on gender diversity, not medical diagnosis. As my article also points out, the SoC concede there is no evidence that it is possible to safely and reliably differentiate between psychotic and non-psychotic causes of gender diversity.^2^

Among the many ways this is reflected in practice is that the only mention of psychosis in the clinical guidelines used by all Australia’s paediatric gender clinics is that it ‘should not necessarily prevent medical transition in adolescents with gender dysphoria’.^2^ Publication of the internal communications of the authors of the SoC revealed that they did not consider dissociative identity disorder or homelessness to be contraindications to hormone therapy or orchiectomy in gender-diverse patients.^3^

Even more concerning is the revelation that Victoria Health has now dispensed with the need for specialist mental health screening at all, and they are training hundreds of GPs to initiate hormone therapy for paediatric gender medicine patients without input from a psychiatrist.^4^ All of these examples demonstrate how the assumption that there are no pathological aetiologies of gender diversity prevents the assessment of pathological aetiologies in individual gender-diverse patients.

Finally, while Erasmus refers to a systematic review from 2023 to claim that the GAMOC is supported by evidence, the comprehensive Cass Review released in April this year definitively concludes that there is no high quality evidence that the GAMOC improves paediatric gender patients’ health or mental health.^5^
